# Maternal and paternal support for physical activity and healthy eating in preschool children: a cross-sectional study

**DOI:** 10.1186/s12889-015-2318-9

**Published:** 2015-09-28

**Authors:** Stephanie Schoeppe, Stewart G. Trost

**Affiliations:** Centre for Children’s Health Research, Institute of Health and Biomedical Innovation, Queensland University of Technology, 62 Graham Street, South Brisbane, QLD 4101 Australia; School of Exercise and Nutrition Sciences, Institute of Health and Biomedical Innovation, Queensland University of Technology, 60 Musk Avenue, Kelvin Grove, Brisbane, QLD 4059 Australia

**Keywords:** Social support, Mother, Father, Diet, Active play, Cross-sectional, Survey

## Abstract

**Background:**

Parental support is a key influence on children’s health behaviours; however, no previous investigation has simultaneously explored the influence of mothers’ and fathers’ social support on eating and physical activity in preschool-aged children. This study evaluated the singular and combined effects of maternal and paternal support for physical activity (PA) and fruit and vegetable consumption (FV) on preschoolers’ PA and FV.

**Methods:**

A random sample comprising 173 parent–child dyads completed validated scales assessing maternal and paternal instrumental support and child PA and FV behaviour. Pearson correlations, controlling for child age, parental age, and parental education, were used to evaluate relationships between maternal and paternal support and child PA and FV. K-means cluster analysis was used to identify families with distinct patterns of maternal and paternal support for PA and FV, and one-way ANOVA examined the impact of cluster membership on child PA and FV.

**Results:**

Maternal and paternal support for PA were positively associated with child PA (r = 0.37 and r = 0.36, respectively; *P* < 0.001). Maternal but not paternal support for FV was positively associated with child FV (r = 0.35; *P* < 0.001). Five clusters characterised groups of families with distinct configurations of maternal and paternal support for PA and FV: 1) above average maternal and paternal support for PA and FV, 2) below average maternal and paternal support for PA and FV, 3) above average maternal and paternal support for PA but below average maternal and paternal support for FV, 4) above average maternal and paternal support for FV but below average maternal and paternal support for PA, and 5) above average maternal support but below average paternal support for PA and FV. Children from families with above average maternal and paternal support for both health behaviours had higher PA and FV levels than children from families with above average support for just one health behaviour, or below average support for both behaviours.

**Conclusions:**

The level and consistency of instrumental support from mothers and fathers for PA and FV may be an important target for obesity prevention in preschool-aged children.

## Background

Over the last three decades, the prevalence of overweight and obesity has more than tripled among US children and adolescents [1]. Data from the 2011–2012 National Health and Nutrition Examination Survey (NHANES) indicate that 16.9 % of young people between the ages 2–19 years are obese, with a further 14.9 % considered to be overweight [2]. Preschool-aged children are also affected by the obesity problem. Data from the most recent NHANES indicates that 8.4 % of US children between the ages 2–5 years are obese, with a further 14.4 % considered to be overweight [2]. Obese preschool children are significantly at increased risk for child and adolescent obesity [3] and they are more likely than their non-obese peers to experience significant short- and long-term health problems such as hyperlipidaemia, hypertension, insulin resistance, respiratory problems, and orthopaedic complications [1].

Regular physical activity and proper nutrition play significant roles in the prevention of obesity and related cardio-metabolic disorders, even in young children [3, 4]. Yet, despite these benefits, substantial percentages of preschool-aged children do not meet current recommendations for physical activity, and daily fruit and vegetable consumption. For example, a US study estimated that only one third of 3–5 year olds meet the National Association for Sport and Physical Education (NASPE) physical activity guidelines for preschoolers recommending at least two hours of physical activity daily [5]. Furthermore, less than half of US 2–8 year olds meet the American dietary guidelines recommending that young children eat at least four to five servings of fruits and vegetables every day [6].

Parents can influence their children’s eating and physical activity behaviours by providing instrumental support, such as encouraging children to eat healthy foods and be physically active, providing access to healthy foods and physical activity equipment at home, and establishing rules that limit the consumption of unhealthy snacks [7, 8]. Previous research has shown parental support to be positively associated with children’s fruit and vegetable consumption (FV) and physical activity (PA) levels [7, 8]. However, many important questions remain unexplored. First, the majority of studies investigating the association between parental support and children’s obesogenic behaviours have focused on school-aged children [7, 8]. Few investigations have explored the influence of parental support on eating and PA behaviour in preschool-aged children [7, 9]. Second, to date, research related to food and physical activity parenting has almost exclusively focused on mothers. Relatively few studies examining obesogenic behaviours in young children have differentiated between the support provided by mothers and fathers [7, 10]. Third, studies conducted to date have investigated the influence of parental support in a single health domain. To our knowledge, no studies have explored patterns of parental support in multiple domains of health-related behaviour. Importantly, identifying clusters of families that systematically vary on maternal and paternal support for *both* healthy eating and physical activity can inform the development of potentially more effective targeted and tailored parenting interventions.

To address these gaps in the research literature, the present study aimed to: 1) Investigate associations between maternal and paternal instrumental support for PA and FV and preschool children’s PA and FV behaviours; 2) Determine the extent to which these associations are moderated by parent and child gender; and 3) Identify groups of families with distinct configurations of maternal and paternal support for PA and FV and examine their impact on child behaviour.

## Methods

### Sample

Participants for this study were recruited from family day care homes participating in the Healthy Home Child Care Project, a cluster randomised trial to promote healthy eating and regular physical activity in children attending family day care homes [11]. Family day care homes were recruited through five Child Care Resource and Referral (R&R) hubs serving seven economically diverse counties in Oregon, USA. They were randomly selected using the Oregon Child Care R&R Network database as a sampling frame. Prior to selection, the sample was stratified by R&R hub and homes within each stratum were sampled with a probability proportional to the total number of family day care homes operating in the hub. Initially, 63 family day care homes enrolled in the study. Of those, five had too few children under their care when data collection began (less than four children) and two others closed their business before data collection, which left a sample of 56 family day care homes. [11]. All children between the ages 2–5 years attending these family day care homes were invited to take part in the study. The number of eligible children within each home ranged from one to six, with a median of four child participants per family day care home. Data for the current study were collected during baseline assessments which took place between October 2010 and March 2011. Of the 336 children eligible to participate in the study, 303 (90 %) completed baseline assessments for height, weight, and body mass index (BMI). Of this number, 173 (51.5 %) parents or caregivers completed a take-home questionnaire measuring socio-demographic information, parenting practices related to healthy eating and physical activity, and their child’s FV consumption and PA levels. Ethical approval for the Healthy Home Child Care Project was obtained from the Oregon State University Institutional Review Board, and before participating, family day care providers and the children's parents provided written informed consent.

### Measures

#### Maternal and paternal support for physical activity

Maternal and paternal support for children’s PA was assessed using a 5-item scale developed by Sallis et al. [12]. Parents reported how often the mother/female adult and father/male adult in the household would ‘encourage their child to do physical activities or play sports’; ‘play outside or do physical activity or sports with their child’; ‘provide transportation to a place their child can do physical activity or play sports’; ‘watch their child participate in sport, physical activities or outdoor games’; and ‘tell their child that physical activity is good for his or her health’. Responses were recorded on six-point scale with endpoints ranging from zero (never) to five (daily). In the current sample, the internal consistency of the scale, as measured by Cronbach’s alpha, was 0.77 and 0.80 for maternal and paternal support, respectively.

#### Maternal and paternal support for fruit and vegetable consumption

Maternal and paternal support for children’s FV was measured using a five-item scale developed by Rosenkranz et al. [13]. Parents reported how often the mother/female adult or father/male adult in the household would ‘prepare or cut up vegetables and have them available in the refrigerator for their child’; ‘have prepared or cut-up fruits available for their child’s snack’; ‘encourage their child to eat fruits or vegetables’; ‘eat fruits and vegetables with their child, or prepare/cook fruits and vegetables with their child’; and ‘tell their child that eating fruits and vegetables is good for their health’. Responses were recorded on a six-point scale with endpoints ranging from zero (never) to five (daily). In the current sample, the internal consistency of the scale, as measured by Cronbach’s alpha was 0.77 and 0.82 for maternal and paternal support, respectively.

#### Child physical activity

Children’s PA was measured using the Burdette outdoor playtime checklist [14]. Parents reported the amount of time their child spent playing ‘in the yard or street around the house’ and ‘at a park, playground, or outdoor recreation area’ on a typical day in the last month. For each of these locations, the day was segmented into three time periods: wake-up time until noon, noon until six PM, and six PM until bedtime. Within each time period, the amount of outdoor playtime was reported using a five-point scale with the following responses: 0 min, 1–15 min, 16–30 min, 31–60 min, and over 60 min. Responses for each time interval were coded zero through four (0 = 0 min, 1 = 1 – 15 min, 2 = 16–30 min, 3 = 31–60 min, 4 = over 60 min) and summed over both locations to provide an activity index ranging from 0–24. The Burdette outdoor playtime checklist has shown acceptable validity (*r* = 0.33) against accelerometry for the assessment of overall physical activity in preschool children [14].

#### Child fruit and vegetable consumption

Children’s FV consumption was measured using items from the US School Physical Activity and Nutrition Survey [15]. Parents were asked ‘how many servings of fruit’ and ‘how many servings of vegetables’ does your child eat on a typical day. To help parents estimate the number of servings, pictures and standard definitions of fruit and vegetable serving sizes were included in the survey (e.g., one medium piece of fresh fruit, one small bowl of green salad, ½ cup/4 oz of fresh or cooked vegetables). Response categories were zero, one, two, three and four or more servings per day. Responses to both items were summed to provide an index of daily FV consumption. The items have evidence of acceptable test-retest reliability (*r* = 0.73-0.79) and validity compared to a 24-h recall (*r* = 0.53-0.57) [16].

### Statistical analyses

Partial Pearson correlations were used to evaluate associations between maternal and paternal support for PA and FV and children’s PA and FV behaviour, controlling for child age, parental age, and parental education. Partial correlations were calculated for the entire sample and by gender. To identify groups of families with distinct configurations of maternal and paternal support for the target health behaviour, standardized scores on the maternal and paternal support for PA and FV scales were entered into a cluster analysis. To determine the possible number of clusters in the data, we first performed a hierarchical cluster analysis using Ward’s method and the squared Euclidian distance as a proximity measure. The number of clusters was based on the proximity matrix, fusion coefficients from the agglomeration schedule, and inspection of the dendrogram and icicle plots. A plot of the within groups sum of squares by number of clusters was also used to determine the appropriate number of clusters [17]. After determining the number of clusters to test, we then completed a k-means cluster analysis to: 1) estimate initial cluster means; 2) determine cluster membership across the entire sample; and 3) estimate the final solution for the cluster means. In k-means clustering, k well-spaced observations from the data are selected at random and used as the initial estimate of the cluster means. Cases are then assigned to the nearest cluster based on its distance from the initial cluster means. After assigning all cases to a cluster, the cluster means are recomputed on the basis of its member cases, and the assignment of cases to the nearest cluster is repeated. The iterative process is repeated until no more cases move from one cluster to another or the stated maximum number of iterations is reached. To investigate the effects of cluster membership on child behaviour, between-cluster differences on child PA and FV consumption were tested for significance using one-way ANOVA with Fisher LSD post-hoc comparisons. Both the cluster analyses and between-cluster comparisons were completed using standardised data (sample-based z-scores). All analyses were performed in IBM SPSS Statistics (version 21.0).

## Results

Descriptive data for the 173 parent–child dyads are shown in Table 1. The majority of respondents (N = 135, 78 %) came from households with two parents or adult caregivers in the home. Of these households, 127 (94.0 %) of the questionnaires were completed by the mother or adult female caregiver. There were no significant differences between the 173 children with parent questionnaire data and the 130 children with only height and weight assessments with respect to gender (52.6 % male), age (3.3 ± 1.2 y), BMI (16.7 ± 1.6), and BMI percentile (62.6 ± 24.9). Descriptive statistics for the maternal and paternal support variables, and child PA and FV behaviour are presented in Table 2. On average, mothers and fathers reported greater support for FV than PA, with mothers reporting more support than fathers for both behaviours. Children’s mean daily PA score was 11.8 (SD = 0.4), and their mean daily FV was 4.9 (SD = 0.1) servings a day.

**Table 1:** Socio-demographic statistics for the 173 parent child dyads

Parents	
Maternal age^a^	
<25	8.1
25–39	78.4
≥40	13.5
Paternal age^a^	
<25	2.9
25–39	70.8
≥40	26.3
Maternal education^a^	
High school	16.6
College graduate	69.2
Postgraduate degree	14.2
Paternal education^a^	
High school	22.7
Graduate college	62.4
Postgraduate degree	14.9
Children	
Male^a^	50.9
Age in years^b^	3.3 (0.1)
Non-Hispanic White^a^	94.2
BMI percentile^b^	69.5 (25.6)

^a^%

^b^Mean (SD)

**Table 2:** Mean (SD) scores for the maternal and paternal support variables and child behaviour

	All children	Boys	Girls
Maternal support			
Physical activity^a^	3.1 (0.1)	3.1 (0.1)	3.1 (0.1)
Fruit/vegetable consumption^a^	4.0 (0.1)	4.0 (0.1)	4.0 (0.1)
Paternal support			
Physical activity^a^	2.9 (0.1)	3.1 (0.1)	2.7 (0.1)
Fruit/vegetable consumption^a^	3.3 (0.1)	3.4 (0.1)	3.2 (0.2)
Child behaviour			
Daily physical activity^b^	11.8 (0.4)	11.7 (0.5)	11.8 (0.5)
Daily fruit/vegetable consumption^c^	4.9 (0.1)	4.8 (0.2)	4.9 (0.2)

There were no significant differences by child gender

^a^Scores ranged between 0–5

^b^Scores ranged between 0–24

^c^Scores ranged between 0–8

The results of the partial correlation analyses are reported in Table 3. Across the entire sample, maternal and paternal support for PA were positively associated with child PA (r = 0.37 and r = 0.36, respectively; *P* < 0.001). Correlations were moderate in both boys and girls; however, maternal and paternal support for PA were more strongly correlated with PA in girls (r = 0.45 and r = 0.45, respectively; *P* < 0.001) than in boys (r = 0.31 and r = 0.26, respectively; *P* < 0.05). Across the entire sample, maternal support for FV was positively correlated with child FV consumption (r = 0.35; *P* < 0.001). This correlation was significant in both boys and girls, albeit its strength was moderate in girls (r = 0.46; *P* < 0.001) and small in boys (r = 0.26; *P* < 0.05). In contrast, paternal support for FV was not associated with child FV consumption when calculated for the entire sample (r = 0.13; *P* = 0.16) or girls alone (r = 0.05; *P* = 0.68); however, the correlation among boys just failed to reach statistical significance (r = 0.25; *P* = 0.06).

**Table 3:** Correlations between maternal and paternal support, and child physical activity and fruit and vegetable consumption

	Maternal support	Paternal support
Physical activity		
All children	0.37**	0.36**
Boys	0.31**	0.26*
Girls	0.45**	0.45**
Fruit and vegetable consumption		
All children	0.35**	0.13
Boys	0.26*	0.25
Girls	0.46**	0.05

Controlled for child age, and maternal and paternal age and education

**P < 0.001 level

*P < 0.05 level

**Fig. 1:**
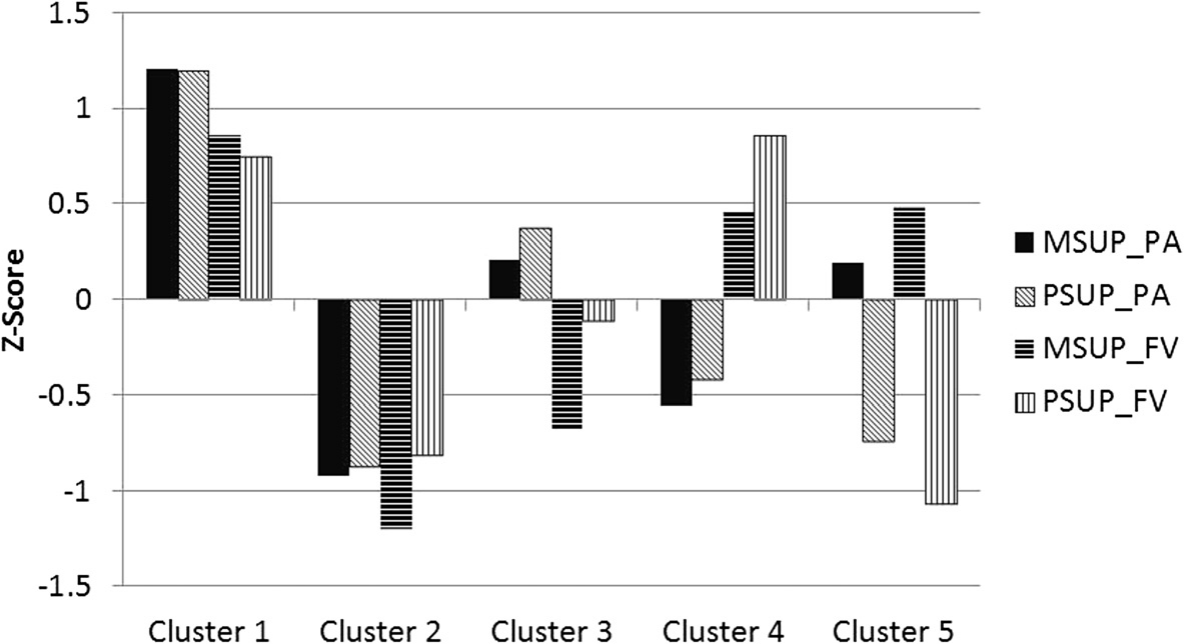
Final cluster centroids for maternal and paternal support for physical activity (PA), and fruit and vegetable consumption (FV). Abbreviations: MSUP_PA = maternal support for physical activity, PSUP_PA = paternal support for physical activity, MSUP_FV = maternal support for fruit and vegetable consumption, PSUP_FV = paternal support for fruit and vegetable consumption

After list-wise deletions for missing maternal or paternal support and/or child health behaviour data, the effective sample size for the cluster analysis was N = 128. Five clusters best categorised the data; these clusters remained stable in the K-means iterative clustering procedure, and accounted for 61- 69 % of variance in the parental support variables. The final solution for the cluster centroids are shown in Fig. 1. The first cluster was characterised by above average maternal and paternal support for both PA and FV (N = 35). The second cluster was characterised by below average maternal and paternal support for both PA and FV (N = 26). The third cluster was characterised by above average maternal and paternal support for PA, but below average maternal and paternal support for FV (N = 23). The fourth cluster was characterised by above average maternal and paternal support for FV, but below average maternal and paternal support for PA (N = 22). The fifth cluster was characterised by discordant maternal and paternal support, where mothers’ support for PA and FV was above average and fathers’ support for PA and FV was below average (N = 22).

**Fig. 2:**
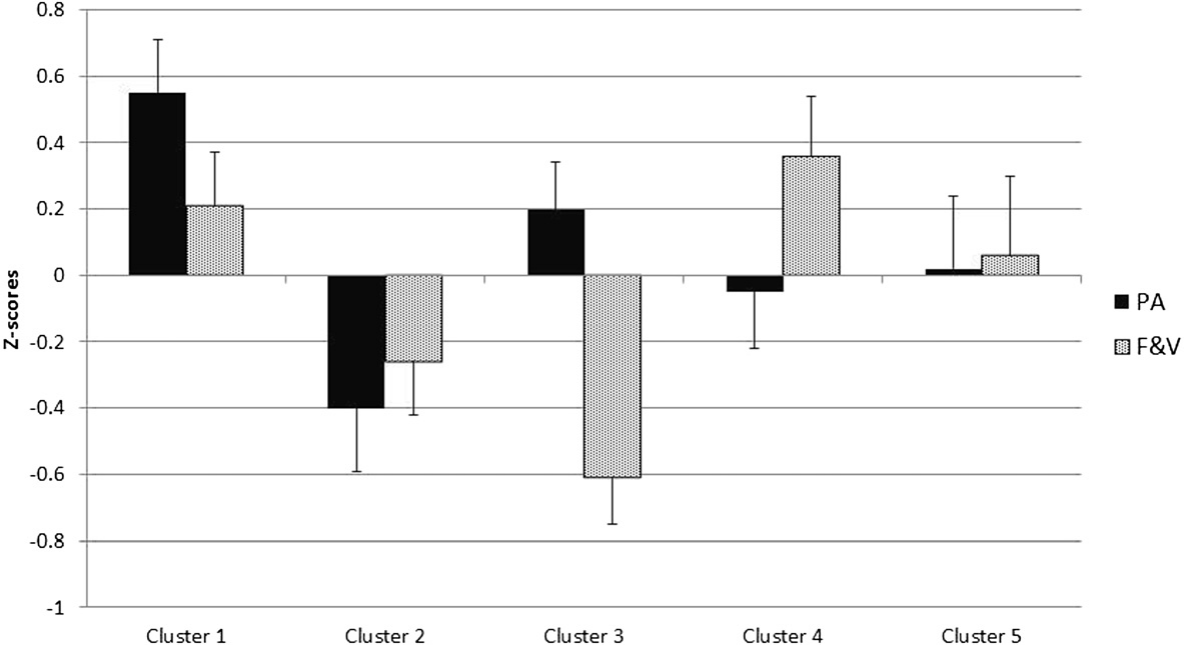
Mean ± SD z-scores for child physical activity (PA), and fruit and vegetable consumption (FV) outcomes across the five parental support clusters

Figure 2 displays the mean z-scores for child PA and FV across the five parental support clusters. The clusters differed significantly on child PA scores (F _4,123_ = 4.55, *P* = 0.002) and FV consumption (F _4,123_ = 5.15, *P* = 0.001). PA scores for clusters one and three (high maternal and paternal support for PA) were significantly higher than PA scores for clusters two, four and five (difference range = 0.52 – 0.94, *P* < 0.05). FV scores for clusters one and four (high maternal and paternal support for FV) were significantly higher than those for clusters two and three (difference range = 0.52 – 0.98, *P* < 0.05). PA and FV scores for cluster five (high maternal support and low paternal support) tended to be higher than scores from clusters with below average support from both parents, and lower than scores from clusters with above average support from both parents.

## Discussion

To our knowledge, this is first study to investigate the differential influence of maternal and paternal instrumental support on preschoolers’ activity and eating behaviours. The results of the correlation analyses showed that both maternal and paternal support for PA were positively associated with children’s PA behaviour. In contrast, maternal but not paternal support for FV was positively associated with children’s FV behaviour. Another unique aspect of this study was the use of cluster analysis to identify familial groups with distinct configurations of maternal and paternal support for PA and FV. Children from families with above average maternal and paternal support on *both* health behaviours had higher PA and FV levels than children from families with above average support on *just one* health behaviour, providing evidence of a possible synergism between food and physical activity parenting practices. Notably, children from families with discordant parental support (high maternal support, low paternal support) had higher PA and FV than children from families with below average support from both parents, but lower than scores from clusters with above average support from both parents, reinforcing the continued need to engage both mothers and fathers in efforts to promote healthy eating and regular PA in children under five.

Maternal and paternal support for PA was positively associated with children’s PA behaviour. This finding is consistent with the results of some [18, 19], but not all [20] studies investigating the influence of parental social support on PA behaviour in preschool children. Loprinzi and Trost [19] found a significant positive association between parental support for PA and preschoolers’ PA at home. Cleland et al. [18] showed that parental support in the form of encouragement for outdoor play was significantly associated with preschoolers’ outdoor activity. In contrast, Pfeiffer et al. [20] found no association between parental support and PA behaviour in preschool children. Although previous studies have identified parental support as a significant influence on preschoolers’ PA, none have distinguished between the instrumental support provided by mothers and fathers. Our study is the first to demonstrate that both maternal and paternal support for PA have a significant positive influence on PA behaviour in preschool children, although the associations were stronger in girls than boys. The observed gender difference is consistent with the findings by Cleland et al. [18] showing that the association between parental encouragement to play outside and children’s outdoor activity was stronger in girls than boys.

Consistent with the results of previous studies [21, 22], maternal support for FV was positively associated with children’s FV behaviour; although the magnitude of the association was substantially greater in girls than in boys. Paternal support for FV, in contrast, was not associated with FV behaviour. Given that mothers, not fathers, typically purchase, prepare and monitor young children’s foods [11, 22], it is perhaps not surprising to find a positive association between maternal but not paternal support.

A strength of this study was the investigation of both maternal and paternal support and its association with multiple health behaviours in preschool-aged children. Moreover, this was the first study to examine the clustering of maternal and paternal support practices for PA and FV in preschool children. Other strengths include the examination of gender differences, adjustment for potential confounders, and the use of reliable and valid measurement scales. This study also had limitations. First, the cross-sectional design precludes inference on casual relationships. Second, the parent–child dyads were recruited solely through family day care homes. Therefore, the results may not be generalisable to the general US population of preschool-aged children and their parents. Third, since parental education, an indicator of socio-economic status, was collected solely through the parent survey, we could not explore potential socio-economic status differences between children with parent survey data and children without survey data. Moreover, given that most of the questionnaires were completed by the mother or adult female caregiver, we cannot exclude the possibility of social desirability or recall bias. Finally, the reliance on self-reported PA data is a limitation of this study. Although the Burdette outdoor playtime checklist is a validated parent-proxy measure of physical activity in preschool-aged children [14], it does not provide time estimates of physical activity but an activity index ranging from 0 to 24, reflecting children’s relative participation in physical activity. Future studies should consider the use of direct observations or objective measures for assessing child and parent health behaviours.

## Conclusions

Overall, both maternal and paternal support for PA were positively associated with children’s PA. In contrast, maternal but not paternal support for FV was positively associated with children’s FV. In addition, children’s PA and FV levels are higher when both parents provide support for not one but both behaviours. This observation suggests that interventions to promote PA and healthy eating in young children include strategies that address the level and consistency of instrumental support provided by mothers and fathers.

## Abbreviations

PA: Physical activity
FV: Fruit and vegetable consumption
BMI: Body mass index
MSUP_PA: Maternal support for physical activity
PSUP_PA: Paternal support for physical activity
MSUP_FV: Maternal support for fruit and vegetable consumption
PSUP_FV: Paternal support for fruit and vegetable consumption
NHANES: National Health and Nutrition Examination Survey
NASPE: National Association for Sport and Physical Education
ANOVA: Analysis of variance
LSD: Least significant difference
